# Appropriate time to radical surgery for colorectal cancer patients complicated with newly onset cerebral infarction: a propensity score matching analysis

**DOI:** 10.1038/s41598-023-31988-9

**Published:** 2023-03-23

**Authors:** Weidong Dou, Tao Liu, Hang Zheng, Shuo Feng, Yingchao Wu, Xin Wang

**Affiliations:** Department of General Surgery, Peking University First Hospital, Peking University, 8 Xi ShiKu Street, Beijing, 100034 People’s Republic of China

**Keywords:** Cancer, Oncology

## Abstract

The purpose of our study was to compare the short-term outcomes of early (within 3 months after stroke) and nonearly (more than 3 months after stroke) radical colorectal cancer surgery to find an appropriate time to surgery for these colorectal cancer patients complicated with new-onset cerebral infarction. A retrospective analysis of patients with stroke who underwent curative colorectal cancer surgery between January 2010 and December 2020 was conducted. Propensity score matching (PSM) analysis was performed to overcome patient selection bias between the two groups. A total of 395 patients were reviewed. After PSM, 40 patients in the early group and 40 patients in the nonearly group were compared. The median time to surgery was 4 weeks in the early group. The overall incidence of postoperative complications between the groups was not significantly different (*p* = 0.745). The early group was associated with less intraoperative blood loss (50 vs. 100, *p* = 0.029 ml), with no difference in 30-day morbidity and mortality. Additionally, multivariate logistic regression analysis showed that previous abdominal surgery (*p* = 0.049) was an independent risk factor for postoperative complications after matching. Before matching, multivariate logistic analysis showed that ESRS (*p* = 0.028) and MRS (*p* = 0.039) were independent risk factors. Radical surgery after 4 weeks of cerebral infarction may be feasible for colorectal cancer patients with new onset stroke, as it appear not to increase the perioperative complications of Clavien–Dindo grade II or higher, while strengthening the preoperative evaluation and perioperative monitoring.

## Introduction

It is well known that the incidence of most malignancies and strokes increases significantly with age. Colorectal cancer has become the third most commonly diagnosed cancer and the second most common cause of cancer mortality worldwide^1^. Meanwhile, cerebral infarction ranks as the second leading cause of death worldwide^2^. With the advancement of medical treatment, the mortality rate of patients with acute cerebral infarction is decreasing, and an increasing number of patients with cerebral infarction are surviving. Recent studies have found that people with cancer have a significantly higher risk of cerebral infarction than the normal population^3–5^. Predictably, there will be an increasing number of colorectal cancer patients complicated with cerebral infarction. Therefore, it is of great positive significance to carry out clinical research on people suffering from colorectal cancer complicated with cerebral infarction.

Clinically, patients newly diagnosed with cancer often ask about the timing of curative surgery and the acceptable delay between diagnosis and surgery. In addition, patients and providers are often concerned that the longer they wait for surgery, the more anxious the patient becomes and the greater the risk of tumor mutation, metastasis and worse outcomes. However, there are no guidelines or related research reports on the time to radical surgery for colorectal cancer patients with recent cerebral infarction. The present study compared the short-term outcomes in a single-center cohort of patients with cerebral infarction who underwent radical resection for colorectal cancer within 3 months and those who underwent surgery more than 3 months after cerebral apoplexy, and this study aimed to find an appropriate time to surgery.

## Materials and methods

### Patients

**Figure 1:**
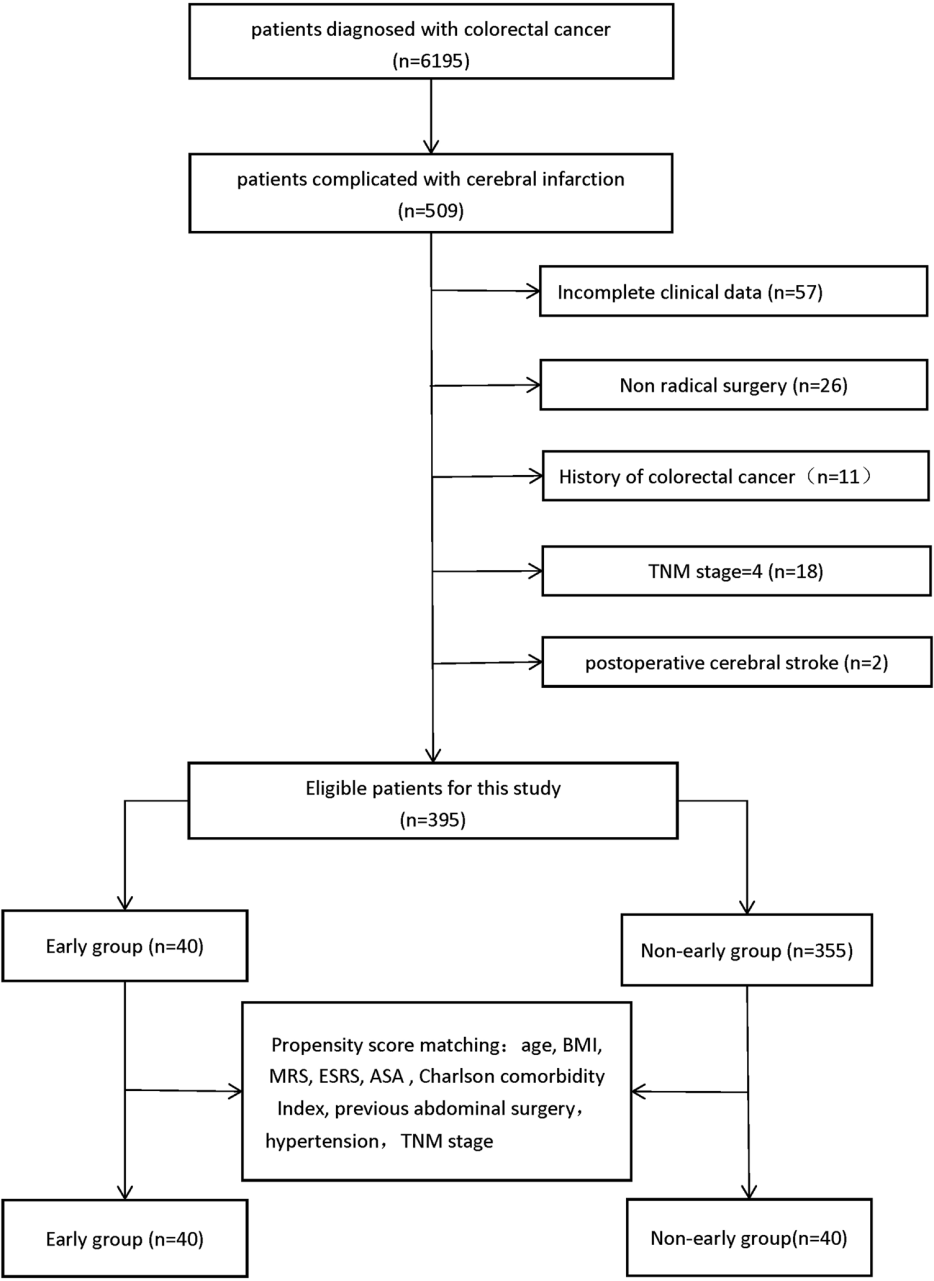
The experimental protocol was approved by the ethics committee of the Peking University First Hospital. The study did not require informed consent from the patients, as it was a retrospective analysis.

The clinical data of patients were obtained from a prospectively collected database and electronic patient record system at the Department of General Surgery, Peking University First Hospital. We initially selected 6195 patients with CRC who were treated with surgery between January 2010 and December 2020. Then, 508 patients with cerebral apoplexy were screened and divided into an early group (radical surgery within 3 months) and a nonearly group (radical surgery after 3 months, Including colorectal cancer patients who have a new cerebral infarction and wait for radical surgery for more than 3 months and those have a history of cerebral infarction for more than 3 months) according to the time of surgery after stroke. The exclusion criteria were as follows: (1) patients with incomplete clinical data, (2) patients with postoperative cerebral infarction rather than preoperative (3) patients with stage IV, (4) patients who did not undergo radical surgery, and (5) patients with a previous history of colorectal cancer. After screening, eligible patients with CRC and cerebral infarction were identified. A flowchart is shown in Fig. 1.

### Data collection

The following data were collected and analyzed for each patient: (1) The preoperative patient information including age, sex, body mass index (BMI), concentration of albumin, hemoglobin and platelets, American Society of Anesthesiology classification score (ASA), and Charlson comorbidity index score. Hypertension, previous abdominal surgery, time to surgery (TTS) (2) stroke-related scores: Essen Stroke Risk Score (ESRS), Modified Rankin Scale (MRS) (3)tumor-related information including tumor location, TNM stage; (4) Coagulation function related indicators: International Normalized Ratio (INR), d-dimer; and (5) surgical treatment information including surgical type and surgical approach, operative time, intraoperative blood loss. (6)The postoperative outcomes included the short-term postoperative complications (within 30 days after surgery), postoperative hospital stay, intensive care unit (ICU) stay, whether a ventilator was used, and time to first flatus. The postoperative complications were defined by the Clavien–Dindo classification (CDC), and those classified as ≥ grade II were included in this analysis. Anastomotic leakage was identified by clinical or radiological evidence of anastomotic suture dehiscence, with or without the need for surgical exploration; postoperative ileus was defined as a delay in the time of first flatus or abdominal distention and vomiting after eating, confirmed by an upright abdominal plain film or an abdominal CT.

### Propensity score matching analysis

To minimize bias caused by the baseline characteristics of patients, we performed propensity score matching (PSM) analyses due to the retrospective nature of the analysis^6–8^. We selected the following factors as covariates for PSM: age, BMI, MRS, ESRS, ASA, Charlson comorbidity index, previous abdominal surgery, hypertension, and TNM stage. After calculating propensity scores using the multiple logistic regression model, the early and nonearly patients were then paired 1:1 based on these propensity scores by applying the nearest-neighbor algorithm within a caliper of 0.02.

### Statistical analysis

For continuous data, the Kolmogorov–Smirnov test was used to assess the distribution uniformity, and the t test or Mann–Whitney test was used depending on the data distribution into the analyzed population. Normally distributed data are described as the mean and standard deviation; otherwise, data are reported as the median and interquartile range (IQR). The chi-squared test and Fisher’s exact test were used for comparing categorical variables. Univariate analysis was utilized to assess potential factors affecting the short-term outcomes. The variables for multivariate logistic regression analysis were MRS and those with a *p* value < 0.1 after univariate analysis. The baseline characteristics and operative and postoperative variables were compared using bivariate analysis. All tests were two-sided, and a *p* value < 0.05 was considered statistically significant. All statistical analyses and PSM were performed using SPSS (version 26.0; IBM, Armonk, NY, USA).

### Methods statement

All methods were carried out in accordance with relevant guidelines and regulations.

### Informed consent statement

Informed consent waiver is approved by the ethics committee of the Peking University First Hospital.

## Results

### Patient characteristics

From January 2010 to December 2020, 395 patients with colorectal cancer and stroke met the inclusion criteria. The patients were divided into two groups according to the interval to surgery after cerebral infarction. Forty patients were included in the early group, and 355 patients were included in the nonearly group. The characteristics of the original and matched patients are displayed in Table 1. Before PSM, the early and nonearly groups differed significantly in ESRS (*p* = 0.01) and MRS (*p* < 0.001). After PSM, the study population was composed of 80 patients: 40 in the early group and 40 in the nonearly group. There were no significant differences between the groups with regard to the baseline demographic and clinical variables.

**Table 1:** The comparison of patient clinical characteristics before and after propensity score matching.

Characteristic	Patients before PSM				Patients after PSM			
	Early group (n = 40)		Non-early group (n = 355)	*p*	Early group (n = 40)	Non-early group (n = 40)		*p*
Sex (male/female)	29/11		228/127	0.382	29/11	21/19		0.065
Diagnosis n (%)				0.603				0.099
Right colon cancer	11 (27.5%)		95 (26.8%)		11 (27.5%)	10 (25%)		
Left colon cancer	1 (2.5%)		24 (6.7%)		1 (2.5%)	7 (17.5%)		
Rectal cancer*	28 (70%)		236 (66.5%)		28 (70%)	23 (57.5%)		
Hypertension n (%)	14 (35%)		145 (40.8%)	0.502	14 (35%)	12 (30%)		0.633
Surgery n (%)				0.875				0.145
LS colectomy	6 (15%)		50 (14.1%)		6 (15%)	3 (7.5%)		
open colectomy	6 (15%)		62 (17.5%)		6 (15%)	13 (32.5%)		
LS dixon/Miles	15 (37.5%)		148 (41.6%)		15 (37.5%)	17 (42.5%)		
Open dixon/Miles	13 (32.5%)		95 (26.8%)		13 (32.5%)	7 (17.5%)		
ESRS n (%)				**0.001**				0.127
Low (1–3)	15 (37.5%)		161 (45.4%)		15 (37.5%)	12 (30%)		
Middle (4–6)	22 (55%)		194 (54.6%)		22 (55%)	28 (70%)		
High (7–9)	3 (7.5%)		0		3 (7.5%)	0		
MRS n (%)				0.803				1.000
0–1	34 (85%)		310 (87.3%)		34 (85%)	34 (85%)		
2–3	6 (15%)		45 (12.7%)		6 (15%)	6 (15%)		
ASA n (%)				**0.034**				0.945
2	9 (22.5%)		139 (39.2%)		9 (22.5%)	11 (27.5%)		
3	26 (65%)		199 (56.1%)		26 (65%)	24 (60%)		
4	5 (12.5%)		17 (4.7%)		5 (12.5%)	5 (12.5%)		
TNM n (%)				0.166				0.645
I	7 (17.5%)		52 (14.6%)		7 (17.5%)	10 (25%)		
II	20 (50%)		132 (37.2%)		20 (50%)	20 (50%)		
III	13 (32.5%)		171 (48.2%)		13 (32.5%)	10 (25%)		
PAS n (%)	4 (10%)		71 (20%)	0.142	4 (10%)	4 (10%)		1.000
D-dimer n (%)				0.494				0.465
Normal	30 (75%)		228 (64.2%)		30 (75%)	26 (65%)		
High	10 (25%)		123 (34.6%)		10 (25%)	14 (35%)		
Low	0		4 (1.2%)					
INR n (%)				0.671				1.000
Normal	31 (77.5%)		289 (81.4%)		31 (77.5%)	30 (75%)		
High	9 (22.5%)		66 (18.6%)		9 (22.5%)	10 (25%)		
CCI n (%)				0.627				0.964
o	17 (42.5%)		164 (46.2%)		17 (42.5%)	17 (42.5%)		
1	17 (42.5%)		119 (33.5%)		17 (42.5%)	15 (37.5%)		
2	3 (7.5%)		46 (13%)		3 (7.5%)	4 (10%)		
≥ 3	3 (7.5%)		26 (7.3%)		3 (7.5%)	4 (10%)		
Age years	75 (66.5–81.8)		72 (65.5–78)	0.198^b^	73.08 ± 11.89	72.58 ± 8.61		0.830^a^
BMI kg/m^2^	24 (20.6–25.6)		23.9 (21.6–26.5)	0.21^b^	23.43 ± 3.33	23.94 ± 3.49		0.501^a^
ALB	39.4 (35.8–42.0)		40.1 (36.4–42.8)	0.409^b^	38.73 ± 4.36	39.41 ± 4.37		0.485^a^
Hgb median (IQR)	116.5 (96.8–146.3)		125 (102–138)	0.629^b^	116.5 (96.8–146.3)	126 (110.3–134.3)		0.490^b^
Plt	215 (174–273.3)		222 (181.3–277)	0.808^b^	237.08 ± 92.45	226.6 ± 80.66		0.591^a^

Significance values are in bold.

*LS* Laparoscopy, *ESRS* Essen stroke risk score, *MRS* Modified Rankin scale, *ASA* American Society of Anesthesiologists, *TNM* tumor-node-metastasis, *PAS* Previous abdominal surgery, *INR* International Normalized Ratio, *CCI* Charlson comorbidity index, *BMI* body mass index, *ALB* albumin, *Hgb* hemoglobin, *Plt* platelets.

*Including Sigmoid cancer.

^a^t test.

^b^Mann–Whitney U test.

### Operative outcomes

The surgical results of the two groups are shown in Table 2. There was a statistically significant difference in the intraoperative blood loss between the two groups, with a median blood loss of 50 ml (20–100) in the early group and 100 ml (50–200) in the nonearly group. The mean operative time and surgical approach in the early group were not significantly different from those in the other group. The median time to surgery was 4 weeks in the early group and 30 months in the nonearly group**.**

**Table 2:** Post-matching operative outcomes.

	Early group (n = 40)	Non-early group (n = 40)	*p* value
Number of patients	40	40	
Operative time ± DS min	209.5 ± 88.0	207.1 ± 70.8	0.891^a^
IBL median (IQR)ml	50 (20–100)	100 (50–200)	**0.029**^b^
Surgical approach n (%)			0.823
Laparoscopy	21 (52.5%)	20 (50%)	
Open	19 (47.5%)	20 (50%)	
Time to surgery*	4 (4–8)W	30 (12–63)M	

Significance values are in bold.

*IBL* intraoperative blood loss.

*W week, *M* Month.

^a^t test.

^b^Mann–Whitney U test.

### Postoperative outcomes and short-term complications

The postoperative outcomes and short-term complications are shown in Table 3. No significant differences were found in terms of the time to first flatus, length of postoperative hospital stay or length of SICU stay between the two groups, with a median time of 4 days versus 4 days, *p* = 0.310; 10 days versus 10 days, *p* = 0.536; and 1 day versus 1.5 days, *p* = 0.224, respectively. Regarding the use of ventilators, the differences were not statistically significant. A total of 11 postoperative events (≥ Clavien–Dindo grade II) occurred in 80 patients (13.8%). The most commonly reported postoperative complication was anastomotic leakage. All anastomotic leaks occurred in the nonearly group. One patient in the nonearly group died of septic shock due to a postoperative intra-abdominal infection. One patient in the early group had weakness and dysarthria, with cranial CT perfusion imaging suggesting a possible new cerebral infarction. Furthermore, we specifically collected postoperative neurological-related complications. There were no statistically significant differences in the overall rate of short-term complications between the early and nonearly groups (12.5% vs. 15%, *p* = 0.745).

**Table 3:** Post-operative outcomes and short-term complications.

	Early group (n = 40)	Non-early group (n = 40)	*p* value
Time to first flatus (days) median (IQR)	4 (3–5)	4 (3–5)	0.310^b^
Postoperative stay (days) median (IQR)	10 (9–14)	10 (9–12)	0.536^b^
SICU stay (days ) median (IQR)	1 (0.25–2)	1.5 (1–2)	0.224^b^
Usage of ventilator n (%)	18 (45%)	22 (55%)	0.371
Types of complication n (%)	5 (12.5%)	6 (15%)	0.745
Anastomotic leakage	0	3 (50%)	
Lymphatic leakage	1 (20%)	1 (16.7%)	
Ileus	0	1 (16.7%)	
Pulmonary embolism	1 (20%)	0	
Cerebral infarction	1 (20%)	0	
Pneumonia	1 (20%)	0	
Anastomotic bleeding	1 (20%)	0	
Mortality	0	1 (16.7%)	
Clavien-Dindo grade ≤ 1 n (%)	3 (7.5%)	0	0.241
Delirium	1	0	
Dysarthria	2	0	

^a^t test.

^b^Mann–Whitney U test.

### Univariate and multivariate logistic regression analyses

The regression analysis results for postoperative complications are shown in Table 4. The univariate analysis indicated that ESRS (*p* = 0.017) was correlated with the postoperative complications. In the multivariate analysis, the results indicated that previous abdominal surgery (*p* = 0.049) was an independent risk factor for postoperative complications. The interval of colorectal cancer surgery after cerebral infarction was not significantly associated with the total postoperative complications in colorectal cancer patients with accompanying cerebral infarction. Since matched patients may not be representative of the whole, we performed univariate and multivariate regression analyses on the 395 patients before matching, and the results are presented in Table 5. Univariate analysis indicated that age (*p* = 0.001), ESRS (*p* < 0.001) and MRS (*p* = 0.006) were correlated with postoperative complications. In the multivariate analysis, the results indicated that ESRS (p = 0.028) and MRS (P = 0.039) were independent risk factors for postoperative complications.

**Table 4:** Post-matching univariate and multivariate logistic regression analysis of postoperative complication.

Variables	Patients without complication	Patients with complication		Univariate analysis *p*		Multivariate analysis *p*
Group n (%)				0.745		
Early	35 (77.5%)	5 (12.5%)				
Non-early	34 (85%)	6 (15%)				
Sex				0.739		
Male	44 (88%)	6 (12%)				
Female	25 (83.3%)	5 (16.7%)				
Diagnosis n (%)				0.551f.		
Right colon cancer	19 (90.5%)	2 (9.5%)				
Left colon cancer	6 (75%)	2 (25%)				
Rectal cancer*	44 (86.3%)	7 (13.7%)				
ESRS				**0.017f.**		0.188
Low (1–3)	26 (96.3%)	1 (3.7%)				
Middle (4–6)	42 (84%)	8 (16%)				
High (7–9)	1 (33.3%)	2 (66.7%)				
Hypertension n (%)				0.324		
Yes	48 (88.9%)	6 (11.1%)				
No	21 (80.8%)	5 (19.2%)				
Surgery n (%)				0.581f.		
Ls colectomy	9 (100%)	0				
Open colectomy	15 (78.9%)	4 (21.1%)				
Ls dixon/Miles	28 (87.5%)	4 (12.5%)				
Open dixon/Miles	17 (85%)	3 (15%)				
MRS n (%)				0.521		0.998
0–1	59 (86.8%)	9 (13.2%)				
2–3	10 (83.3%)	2 (16.7%)				
ASA n (%)				0.635f.		
2	16 (80%)	4 (20%)				
3	44 (88%)	6 (12%)				
4	9 (90%)	1 (10%)				
TNM				0.165f.		0.475
I	16 (94.1%)	1 (5.9%)				
II	36 (90%)	4 (10%)				
III	17 (73.9%)	6 (26.1%)				
PAS n (%)				0.075f.		**0.049**
Yes	64 (88.9%)	8 (11.1%)				
No	5 (62.5%)	3 (37.5%)				
D-dimer n (%)				0.726f.		
Normal	49 (87.5%)	7 (12.5%)				
High	20 (83.3%)	4 (16.7%)				
INR n (%)				0.717f.		
Normal	53 (86.9%)	8 (13.1%)				
High	16 (84.2%)	3 (15.8%)				
CCI n (%)				0.119f.		0.530
0	29 (85.3%)	5 (14.7%)				
1	29 (90.6%)	3 (9.4%)				
2	7 (100%)	0				
≥ 3	4 (57.1%)	3 (42.9%)				
Age years mean ± DS	72.0 ± 10.1	77.7 ± 10.8		0.09^a^		0.339
BMI kg/m^2^ mean ± DS	23.5 ± 3.5	24.6 ± 2.8		0.329^a^		
HGB mean ± DS	120.8 ± 25.8	115.6 ± 24.6		0.534^a^		
ALB mean ± DS	39.1 ± 4.3	39.0 ± 4.8		0.947^a^		
PLT median (IQR)	215 (175–268)	230 (143–281)		0.915^b^		

Significance values are in bold.

*ESRS* Essen stroke risk score, *Ls* Laparoscopy, *MRS* Modified Rankin scale, *ASA* American Society of Anesthesiologists, *TNM* tumor-node-metastasis, *PAS* Previous abdominal surgery, *INR* International Normalized Ratio, *CCI* Charlson comorbidity index, *BMI* body mass index, *ALB* albumin, *Hgb* hemoglobin, *Plt* platelets.

*Including Sigmoid cancer.

^f^Fisher exact test; ^a^t test; ^b^Mann–Whitney U test.

**Table 5:** Pre-matching univariate and multivariate logistic regression analysis of postoperative complication.

Variables	Patients without complication	Patients with complication		Univariate analysis *p*		Multivariate analysis *p*
Gruop n (%)				0.788		
Early	35 (77.5%)	5 (12.5%)				
Non-early	317 (89.3%)	38 (10.7%)				
Sex				1.000		
Male	229 (89.1%)	28 (10.9%)				
Female	123 (89.1%)	15 (10.9%)				
Diagnosis n (%)				0.793f.		
Right colon cancer	96 (90.5%)	10 (9.5%)				
Left colon cancer	23 (75%)	2 (25%)				
Rectal cance*r	233 (86.3%)	31 (13.7%)				
ESRS				** < 0.001f.**		**0.028**
Low (1–3)	167 (94.9%)	9 (5.1%)				
Middle (4–6)	184 (85.2%)	32 (14.8%)				
High (7–9)	1 (33.3%)	2 (66.7%)				
Hypertension n (%)				0.08		0.059
Yes	205 (86.9%)	31 (13.1%)				
No	147 (92.5%)	12 (7.5%)				
Surgery n (%)				0.731f.		
LS colectomy	51 (92.7%)	4 (7.3%)				
Open colectomy	59 (86.8%)	9 (13.2%)				
LS dixon/Miles	145 (89.5%)	17 (10.5%)				
Open dixon/Miles	95 (88%)	13 (12%)				
MRS n (%)				**0.002**		**0.021**
0–1	313 (86.8%)	31 (13.2%)				
2–3	39 (83.3%)	12 (16.7%)				
ASA n (%)				0.122f.		0.970
2	137 (92.6%)	11 (7.6%)				
3	197 (87.6%)	28 (12.4%)				
4	18 (81.8%)	4 (18.2%)				
TNM				0.951		
I	52 (88.1%)	7 (11.9%)				
II	136 (89.5%)	16 (10.5%)				
III	164 (89.1%)	20 (10.9%)				
PAS n (%)				**0.046**		0.054
Yes	290 (90.6%)	30 (9.4%)				
No	62 (82.7%)	13 (17.3%)				
D-dimer n (%)				0.726f.		
Normal	232 (89.9%)	26 (10.1%)				
High	117 (88%)	16 (12%)				
Low	3 (75%)	1 (25%)				
INR n (%)				0.450		
Normal	287 (89.7%)	33 (10.3%)				
High	65 (86.7%)	10 (13.3%)				
CCI n (%)				0.054		0.447
0	164 (90.6%)	17 (9.4%)				
1	123 (90.4%)	13 (9.6%)				
2	44 (89.8%)	5 (10.1%)				
≥ 3	21 (72.4%)	8 (27.6%)				
Age years median (IQR)	72 (65–78)	77 (70–82)		0.001^b^		0.149
BMI kg/m^2^median (IQR)	23.7 (21–26.2)	24.6 (21.6–27.1)		0.343^b^		
HGB median (IQR)	123 (101–139)	123 (100–135)		0.838^b^		
ALB median (IQR)	39.8 (36.4–42.9)	40.3 (36.2–42.2)		0.499^b^		
PLT median (IQR)	222 (179–277)	230 (185–273)		0.808^b^		

Significance values are in bold.

*ESRS* Essen stroke risk score, *Ls* Laparoscopy, *MRS* Modified Rankin scale, *ASA* American Society of Anesthesiologists, *TNM* tumor-node-metastasis, *PAS* Previous abdominal surgery, *INR* International Normalized Ratio, *CCI* Charlson comorbidity index, *BMI* body mass index, *ALB* albumin, *Hgb* hemoglobin, *Plt* platelets.

*Including Sigmoid cancer.

^f^Fisher exact test, ^a^t test, ^b^Mann–Whitney U test.

## Discussion

As a complication of cancer, stroke increases the morbidity and mortality among cancer patients, leading to increased disability and healthcare costs^9^. To date, various studies have discussed the treatment and short-term prognosis of recent stroke patients complicated with colorectal cancer^10–12^. However, there is no universal recommendation for how long a patient with newly diagnosed colorectal cancer and newly onset stroke can safely wait from the time of diagnosis to definitive surgery. A study that included 514,103 colon cancer patients showed an increased mortality risk with time to surgery > 30 days or within the first week compared to surgery performed 7–30 days after diagnosis. Meanwhile, the risk ratio increased with prolonged TTS^13^. Therefore, choosing an appropriate timing of surgery is crucial for patients with colorectal cancer combined with recent cerebral infarction.

The total postoperative outcomes and short-term complications did not differ significantly between the early and nonearly groups, suggesting that early surgery after stroke did not increase the incidence of complications that were above a Clavien–Dindo classification grade II. However, a statistically significant increase in the intraoperative blood loss was found in the nonearly group compared with the early group. There was no significant difference in the duration of surgery or the percentage of patients undergoing laparoscopic surgery between the two groups, so it may not be the surgeon’s surgical skill that accounts for the difference. Considering that the nonearly group was treated with cerebral infarction earlier and took anticoagulants for a longer time, the high amount of intraoperative blood loss may be associated with this.

According to the literature, postoperative pulmonary complications (PPCs) are still relatively common, and the reported incidence of PPCs after colorectal surgery varies from 8 to 20%^14–16^, accounting for over 60% of the postoperative in-hospital deaths^14^. Elderly individuals are associated with decreased lung elasticity and compliance, increased functional residual capacity, and decreased oxygen reserve function. Pain stimulation and the effect of perioperative anesthetic drugs cause shallow and slow breathing, providing conditions for the occurrence of PPCs. Additionally, in elderly patients with cerebral infarction, the cough reflex and ciliary clearance ability of the airway wall were weakened, and the sputum discharge function was reduced, which can both easily cause the occurrence of aspiration. Considering the above factors, preoperative lung function and perioperative arterial blood gas, the anesthesiologist in our hospital has no high tendency to withdraw the tracheal intubation for the colorectal cancer patients with cerebral infarction at the end of the operation. Instead, the doctor in the intensive care unit decides the timing of withdrawal after the patient’s condition in the intensive care unit is stable. Our results demonstrate a low incidence of PPCs, both pre- and postmatch, which may be attributable to preoperative cough and expectoration training, the improvements in chest physical therapy, and nebulization therapy^17–19^.

Postoperative paralytic ileus (POI) is a common postoperative complication after colorectal surgery that increases the hospital stay length by 25%, hospital cost by 15%, and postoperative 30-day complication-associated mortality and affects the patient’s long-term quality of life^20^. Studies have shown that the patient’s postoperative gastrointestinal motility is mainly related to three mechanisms: the neural reflex, inflammatory response and drug effect^21–23^. The reported incidence of POI varies, with some reports indicating a considerably high incidence^23,24^. In our study, although intestinal obstruction, confirmed by imaging, was rare, the time to first flatus was significantly prolonged, with a median time of 4 days. This may be related to the fact that elderly patients with cerebral infarction are more frail and stay in bed for a longer time after surgery. Furthermore, the metabolism of anesthetics and sedatives as well as the recovery of gastrointestinal nerve function is slower postoperatively.

We noted that patients in the early surgery group had radiographically confirmed recurrent cerebral infarction and neurological-related symptoms, even if there was no significant difference between the two groups. It is reported that the incidence of perioperative stoke in noncardiac and nonmajor vascular surgery ranges from 0.1 to 1.9%^25^. In the nonsurgical setting, the stroke mortality is 12.6%^26^. However, in the perioperative setting, the impact of stroke is much higher. Perioperative stroke not only imparts a higher mortality but also increases morbidity^27^. In addition, at 6 months after hospital admission, 43.2% of elderly patients with delirium remained in some form of institutional care^28^. Considering the risk of a new cerebral infarction, it is necessary to communicate the condition in detail with the family before surgery and strengthen the perioperative monitoring.In our medical center, due to concerns about a high risk of bleeding,patient usually started to discontinue anti-thrombotic drugs 5–7 days before surgery and received bridging therapy with low-molecular-weight heparin. Low molecular weight heparin was discontinued from 12 h pre-surgery to 24 h post-surgery. It is well known that advanced age, malignant tumor and long postoperative bed rest are the high risk factors for postoperative thrombosis. Therefore, we will continue to use low-molecular-weight heparin 24 h after surgery without significant bleeding until our patient's postoperative activity gradually recovers, and his oral food intake and other daily activities are well before resuming oral antiplatelet agents.

There are several limitations to this study. First, only 15% of the patients in the early surgery group had residual disability based on MRS, so more data may be needed to confirm that early surgery does not increase the number of complications compared with nonearly surgery for patients with higher MRS. Second, this study was not prospectively conducted and patients were not randomly allocated, In this context, some patients who declined surgery because of comorbidities or high surgical risk, or who had incomplete follow-up data, were unable to be included in our study, leading to selection bias that may have affected the results of our study.Last,Although PSM was used to balance the baseline characteristics of patients, other confounding factors cannot be ruled out as this was a retrospective study..Therefore, further validation in multicenter and prospective randomized studies is needed.

## Conclusion

With enhanced preoperative evaluation and perioperative monitoring, 4 weeks post-stroke may be a viable time for radical surgery in colorectal cancer patients with new onset of stroke, as it does not seem to increase the perioperative complications in patients with Clavien-Dindo class II or higher.

## Data Availability

The data that support the findings of this study are available from the corresponding author, upon reasonable request.

## Abbreviations

BMI: Body mass index
MRS: Modified rankin scale
ASA: American society of anesthesiology classification score
ESRS: Essen stroke risk score
